# Overexpression of DcR3 and Its Significance on Tumor Cell Differentiation and Proliferation in Glioma

**DOI:** 10.1155/2014/605236

**Published:** 2014-03-05

**Authors:** Suning Huang, Gang Chen, Yiwu Dang, Long-Hua Chen

**Affiliations:** ^1^Departments of Radiation Oncology, Nanfang Hospital, Southern Medical University, 1838 Guangzhou Avenue North, Guangzhou, Guangdong 510515, China; ^2^Department of Radiology, First Affiliated Hospital of Guangxi Medical University, 6 Shuangyong Road, Nanning, Guangxi Zhuang Autonomous Region 530021, China; ^3^Department of Pathology, First Affiliated Hospital of Guangxi Medical University, 6 Shuangyong Road, Nanning, Guangxi Zhuang Autonomous Region 530021, China

## Abstract

*Background.* Overexpression of decoy receptor 3 (DcR3) have been reported in various classes of malignancies. However, its expression and clinicopathological contribution in gliomas has not been fully elucidated. *Objective.* To explore the expression and clinical significance of DcR3 protein in relation to tumor cell differentiation and proliferation in glioma cell lines and tissues. *Methods.* One hundred and twenty-five samples of glioma patients and 18 cases of normal brain tissues were recruited. The expression of DcR3 protein was detected using immunohistochemistry. Tumor differentiation was assessed by histologic characters and the status of glial fibrillary acidic protein (GFAP). Tumor cell labeling indexes (LIs) of Ki-67 and PCNA were also obtained. The relationship between the DcR3 level and clinicopathological features was investigated, including tumor differentiation, LIs, and survival. Meanwhile, the expression of DcR3 protein was also measured in the supernatants of 8 glioma cell lines and glioma cells freshly prepared from 8 human glioblastoma specimens by using western blot. *Results.* The level of DcR3 protein in gliomas was significantly higher than that in normal brain tissues (*P* < 0.01). DcR3 expression showed positive correlations with tumor pathological grade (*r* = 0.621, *P* < 0.01) and negative with GFAP expression (*r* = −0.489, *P* < 0.01). Furthermore, there were positive correlations between DcR3 expression and Ki-67, PCNA LIs (*r* = 0.529, *P* < 0.01; *r* = 0.556, *P* < 0.01). The survival in the DcR3 negative group was 50 ± 1.79 months, longer than that of the DcR3 positive group (48.36 ± 2.90), however, without significance (*P* = 0.149). Different levels of DcR3 could also be detected in the culturing supernatants of all the 8 glioma cell lines and glioma cells freshly obtained from 8 human glioblastoma specimens. *Conclusions.* The overexpression of DcR3 might play a crucial role in the tumorigenesis, differentiation, and proliferation of glioma.

## 1. Introduction

The decoy receptor 3 (DcR3), locus on 20q13, is a member of the tumor necrosis factor receptor (TNFR) superfamily [1–4]. Previously, we have reported the overexpression of DcR3 mRNA and protein in sera or tissues of several human malignancies, including hepatocellular carcinoma, gastric carcinoma, and breast cancer [5–8]. The relationship between DcR3 overexpression and tumor deterioration was also revealed [5–8]. DcR3 has been regarded as an oncogene for these malignancies. However, the contribution of DcR3 on glioma has not been fully elucidated. There have been only 3 studies on the association between DcR3 and glioma. Roth et al. [9] investigated the expression of DcR3 in 29 cases of gliomas (stage II, 11 cases and stage IV, 18 cases) with immunohistochemistry. Arakawa et al. [10] further examined *DcR3* gene amplification, level of DcR3 mRNA, and protein in 46 astrocytic brain tumors by quantitative genomic PCR, quantitative reverse transcription-PCR, and immunohistochemistry, respectively. The study included 6 cases of stage II, 16 cases of stage III, and 24 cases of stage IV of gliomas. No normal brain tissues were covered in the aforementioned 2 studies. Hwang et al. [11] compared the variation of DcR3 in the sera of 17 cases of gliomas before versus after surgery. Thus, the objective of the current study was to clarify the role and clinical significance of DcR3 in glioma. DcR3 protein level was detected in a larger cohort with 125 samples of gliomas and 18 cases of normal brain tissues by using immunohistochemistry. Moreover, the supernatants of 8 glioma cell lines and 8 cases of freshly cultured glioma cells were used to detect the soluble DcR3 level by using western blot. DcR3 protein level was further compared to the tumor differentiated and proliferative status, as well as overall survival.

## 2. Materials and Methods

### 2.1. Tissue Samples

This retrospective study included 125 cases of FFPE gliomas and 18 normal brain tissues. The age of the glioma patients ranged from 19 to 78 years, with a mean age of 54 years. Clinicopathological information was provided from medical records. In the current study, 10 cases were pilocytic astrocytomas (WHO grade I) and 39 cases were grade II, including 16 cases of fibrillary astrocytomas, 14 cases of serous astrocytoma, and 9 cases of oligodendrogliomas. These cases were classified into low-grade group for differentiation (*n* = 49). Grade III included 12 cases of anaplastic astrocytomas and 16 cases of anaplastic oligodendrogliomas, while all the 48 cases of grade IV were glioblastomas. Thus, the high-grade group comprised 76 subjects. Seventy-six patients were followed up to 5*∼*58.2 months. Till the endpoint of follow-up, 10 candidates were dead and overall survival (OS) was calculated. The 18 normal tissues were obtained from decompressive resections of traumatic brain injuries. The age of the normal controls ranged from 15 to 57 years, with a mean age of 34 years. All cases were initial tumorectomies without treatment and randomly selected in the First Affiliated Hospital of Guangxi Medical University, China, between July 2003 and October 2007. The study protocol was approved by the Ethical Committee of the First Affiliated Hospital of Guangxi Medical University. Written informed consent was obtained from the patients and clinicians for the usage of the samples for research. All samples were reviewed and diagnosed by two independent pathologists.

### 2.2. Immunohistochemistry

Immunohistochemistry was performed as previously reported [5–7] with the monoclonal antibody DcR3 (37A565, Santa Cruz Biotechnology Inc., CA, USA, 1 : 300 dilution) and monoclonal antibodies Ki-67, proliferating cell nuclear antigen (PCNA), and glial fibrillary acidic protein (GFAP) (Beijing Zhongshan Jinqiao Inc., Beijing, China). The positive signals of DcR3 and GFAP are located in the cytoplasm and cytomembrane. Negative (−), weakly positive (+), moderately positive (++), and strongly positive (+++) were determined according to the immunodetection of stain intensity and amounts of positive cells by two pathologists (YD and GC), who discussed each case until they reached a consensus [5–7]. All of (+), (++), and (+++) were considered as positive expression. The positive signals of Ki-67 and PCNA are distributed in the nuclei. The labeling indexes (LIs) of Ki-67 and PCNA were calculated with the formula (number of positive cells/total number of the cells ×100%) by counting at least 10 representative visions of high magnification (40 × 40).

### 2.3. Cell Culture

Eight human malignant glioma cell lines (LN-18, LN-229, LN-308, LN-428, U87MG, U251MG, U373MG, and D247MG) were purchased from the American Type Culture Collection (ATCC, Rockville, MD, USA). All cell lines were cultured in Dulbecco's modified essential medium (DMEM, Invitrogen Corp., Grand Island, NY, USA) and supplemented with 10% heat-inactivated fetal bovine serum (Invitrogen Corp., Grand Island, NY, USA), 2 mM glutamine, and gentamicin at 37°C in a humidified incubator with 5% CO_2_. Experiments were performed in triplicate.

### 2.4. Preparation of Primary Glioma Cell Cultures

Preparation of primary glioma cell cultures was performed as reported by Roth et al. [9]. Briefly, human brain tumors were achieved from patients with glioblastoma who underwent tumor resection. After tumor removal, the tissues were placed directly in Petri dishes, minced mechanically, and digested enzymatically using collagenase (1 h, 37°C). Afterwards, the dissociated cells were filtered through 100 *μ*m cell strainers to eliminate cell debris. After centrifugation and lysis of erythrocytes by washing with hypotonic water, the glioma cells were washed and resuspended in full medium of DMEM. Conditioned medium was harvested after no more than 5 passages.

### 2.5. Western Blot

Glioma cells (5 × 10^6^) were cultured in serum-free DMEM for 24 h. The supernatants were harvested and subsequently concentrated by centrifugal filter devices. The supernatants of freshly isolated *ex vivo *glioma cells were prepared accordingly [9]. The procedure of western blot was as reported [12–15]. The protein concentration was detected by the Bio-Rad Bradford protein assay and 25 *μ*g of protein was subjected to SDS-PAGE (12 SDS-acrylamide gel) with a loading buffer containing 80 mM Tris-HCl (ph 6.8), 5% SDS, 10% glycerol, 5 mM EDTA (ph 8), 5% 2-Mercapto Ethanol, 0.2% Bromophenol blue, and 1 mM phenylmethylsulfonyl fluoride. The separated proteins were transferred to PVDF membranes (Bio-Rad) for 2 hrs at 100 mA. The membrane was incubated with a DcR3 mouse monoclonal antibody (ab11930, Abcam, Cambridge, CB4 0FL, UK, 1 : 1000 dilution,) or a *β*-actin antibody (A1978 AC-15 1 : 2000 dilution, Sigma-Aldrich, St. Louis, NV, USA). Primary antibodies were detected with an HRP-conjugated secondary antibody (1 : 4000 dilution, ECL Anti-mouse IgG Peroxidase linked Na 931, Sigma-Aldrich, St. Louis, NV, USA) and finally the membranes were subjected to chemiluminescence detection assay. Cell lysis sample from known DcR3 positive hepatocellular carcinoma cell line HepG2 was used as a positive control for western blot.

### 2.6. Statistical Analysis

SPSS19.0 (Munich, Germany) was used for statistical analysis. Mann-Whitney *U* test and Kruskal-Wallis *H* test were performed to analyze the relationship between DcR3 expression and clinicopathological parameters. Results were representative of three independent *in vitro* experiments. Values were presented as the mean ± standard deviation (SD) for Ki-67 and PCNA LIs. One-Way Analysis of Variance (ANOVA) test and Student's paired *t*-test were used to analyze significance between groups. The Least Significant Difference (LSD) method of multiple comparisons between 2 groups was applied when the probability for ANOVA was statistically significant. Kaplan-Meier and log-rank test were performed for the survival analysis. Statistical significance was determined at a *P* < 0.05 level.

## 3. Results

### 3.1. Relationship between DcR3 Expression and Glioma Differentiation

**Figure 1:**
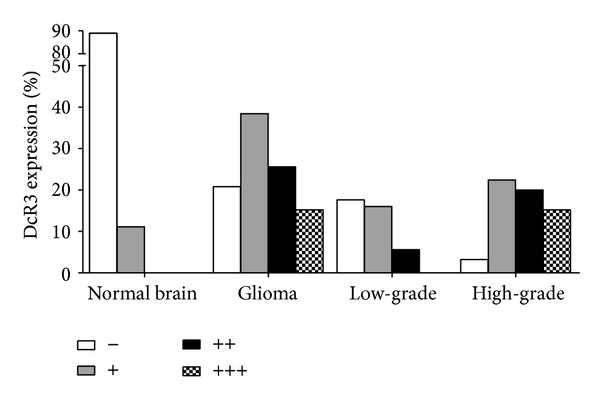
Relationship between DcR3 expression and glioma differentiation.

**Figure 2:**
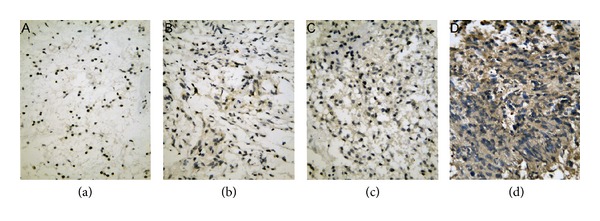
Relationship between DcR3 expression and the grade of glioma: (a) grade I, (b) grade II, (c) grade III, and (d) grade IV (×400).

DcR3 expression was found in 79.2% (99/125) of glioma patients versus 11.1% of normal controls (2/18, *P* < 0.01, Table 1, Figure 1). DcR3 expression was distributed mainly in areas adjacent to ischemic necrosis, especially in the cases of high-grade gliomas (Figure 2). DcR3 expression was associated with the histology grade (*r* = 0.621, *P* < 0.01, Table 1, Figure 1). The positive ratio of DcR3 expression was 55.1% in the group of low-grade (27/49), significantly lower than that of high-grade (94.7%, 72/76, *P* < 0.01). The strongly positive cases with (+++) were only observed in high-grade gliomas. Additionally, a negative relationship was noted between the DcR3 level and GFAP expression (*r* = −0.489, *P* < 0.01), which further indicated the correlation between DcR3 expression and tumor cell differentiation. No relative relationship was found between DcR3 and age or gender (*P* > 0.05, data not shown).

### 3.2. Relationship between DcR3 and LIs of Ki-67 and PCNA

**Figure 3:**
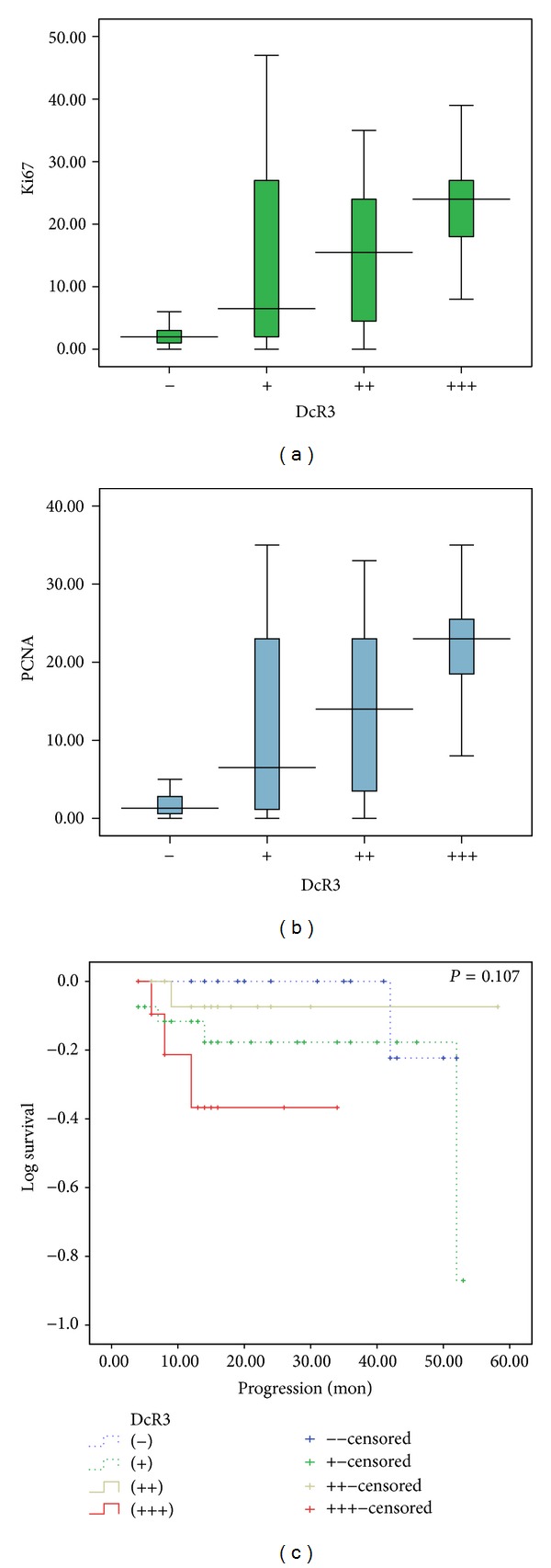
Correlation of DcR3 expressions with proliferation and survival. The relationship between DcR3 expression and labeling indexes (LIs) of Ki-67 (a), PCNA (b), and survival (c).

The LIs of Ki-67 and PCNA were significantly lower in the normal brain tissues as compared to the gliomas. Both LIs increased with the growth of pathology grade (Table 2). There were positive correlations between Ki-67, PCNA LIs, and tumor grade (Ki-67: *r* = 0.728, *P* < 0.01; PCNA: *r* = 0.726, *P* < 0.01). As expected, the negative correlations between the LIs and GFAP were found (Ki-67: *r* = −0.564, *P* < 0.01; PCNA: *r* = −0.568, *P* < 0.01). Ki-67 LI was positively correlated to PCNA LI (*r* = 0.982, *P* < 0.01), however, with a slightly higher value (13.59 ± 1.16 versus 11.84 ± 1.02,   *P* > 0.05). Both LIs of Ki-67 and PCNA were lower in the DcR3 negative group than that in the DcR3 positive group (*P* < 0.01), and they increased with the upregulation of DcR3 expression (Table 2, Figures 3(a) and 3(b)). Thus, there were positive correlations between the LIs and DcR3 expression (Ki-67: *r* = 0.529, *P* < 0.01; PCNA: *r* = 0.556, *P* < 0.01).

### 3.3. Relationship between DcR3 Expression and Survival

In the 76 cases with complete follow-up, the survival of the DcR3 negative group (*n* = 19) was 50.00 ± 1.79 months, slightly higher than that of the DcR3 positive group (*n* = 57, 48.36 ± 2.90 months). However, this difference did not reach the statistical value (*P* = 0.149, data not shown). When looked into the subgroups of DcR3 positive cases, we found that the shortest survival was of 26.29 ± 3.74 months for the strongly positive (+++) group, which was significantly different compared to the DcR3 negative group. However, the *P* value was 0.107 for the comparison of intergroup (Table 3, Figure 3(c)).

### 3.4. Expression of DcR3 in the Supernatants of Glioma Cells

**Figure 4:**
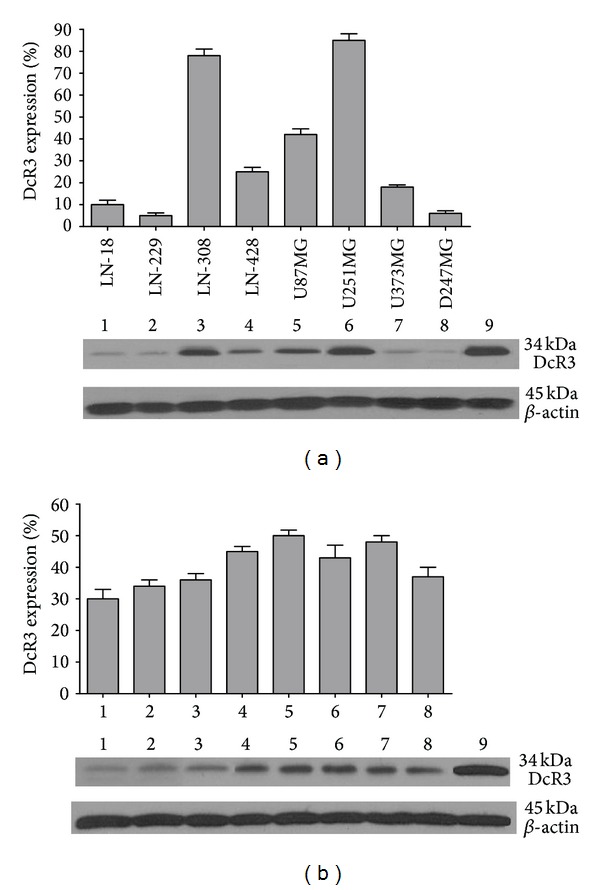
Expression of DcR3 in glioma cells. (a) Supernatants of 8 human long-term glioma cell lines were subjected to western blot; 1: LN-18, 2: LN-229, 3: LN-308, 4: LN-428, 5: U87MG, 6: U251MG, 7: U373G, 8: D247MG, and 9: cell lysis sample from known DcR3 positive hepatocellular carcinoma cell line HepG2. (b) Glioma cells were freshly prepared from human glioblastoma specimens. Supernatants were harvested before the 5th passage and analyzed DcR3 protein by western blot. 1–8 represent 8 individual patients and 9 was positive control as (a).

Western blot showed that DcR3 protein could be detected in all the 8 cell lines tested, with apparently different levels (Figure 4). U251MG and LN-308 had the highest expression of DcR3, whereas almost no DcR3 could be identified in LN-18, LN-229, and D247MG. Glioma cells were also freshly prepared from 8 human glioblastoma specimens. Supernatants were harvested before passage 5 and analyzed for DcR3 for immunoblot. The levels ranged from 30% to 50% as compared to the positive control.

## 4. Discussion

In the current study, we demonstrated that overexpression of DcR3 protein was detected in glioma tissues, as well as in the supernatants of cultural glioma cells. Furthermore, we investigated the relationship between DcR3 level and clinicopathological parameters including tumor differentiation, proliferative status, and patient survival. Our results suggest that DcR3 may act as an oncogene in glioma and could be a potential biomarker for the diagnosis and prognosis for glioma patients.

Previously, we have reported that DcR3 mRNA and protein were highly expressed in HCC, breast cancer, and gastric cancer. The DcR3 level was closely related to the disease progression and tumor metastasis [5–8]. However, the role of DcR3 in glioma has not yet been totally clarified. In the current study, we detected the DcR3 expression in the supernatants of different glioma cell lines, as well as the freshly cultured glioma cells, which confirmed the soluble characteristics of DcR3, consistent with Roth et al. [9]. The aforementioned cell lysis samples were also directly sent to western blot and DcR3 protein could be detected (data not shown). Hwang et al. [11] showed that extremely low level of DcR3 protein was detectable in the serum of glioma patients. However, the preoperative serum concentration of DcR3 in glioma patients was not significantly different from that either in healthy controls or postoperative. The low expression of DcR3 in the serum of glioma patients could be due to the rapid degradation to major circulating metabolic fragment, also the influence of the blood-brain barrier to stop the transfer of DcR3 from brain tissue to the systemic circulation. Concerning the expression of DcR3 in tissues, we found significantly higher expression of DcR3 protein in glioma FFPE tissues than that in the normal brain, which was similar to the status of DcR3 in other malignancies [5, 6, 8, 16–19]. These support the agreement that DcR3 plays a role as an oncogene in various malignant tumors, including gliomas.

Next, we studied the correlation between DcR3 expression and glioma differentiation, which was evaluated by histological features and GFAP staining. Two research groups have reported the relationship between DcR3 level and pathological grade in glioma. DcR3 was found positive in 15 among 18 gliomas of grade IV (83%) by Roth et al. [9], while no DcR3 was detected in 11 cases of grade II. Meanwhile, DcR3 expression was examined in several glioma cell lines and related to the severity of glioma by using immunohistochemistry. Arakawa et al. [10] also studied the expression of DcR3 in 46 cases of glioma. The positive ratio was 16.7% (1/6) for grade II, 0% (0/16) for grade III, and 37.5% (9/24) for grade IV. In the current study, the positive patterns of DcR3 was divided into weakly positive (+), moderately positive (++), and strongly positive (+++). In the low-grade glioma, the DcR3 positive rate was 21.6% (27/125), including 16% weakly expressed cases and 5.6% moderately expressed ones. No strongly expressed samples were identified in this low-grade group, whereas the positive ratio was much higher in the high-grade group (57.6%, *P* < 0.01) with 19 cases being strongly positive expression. There was a positive correlation between DcR3 expression and the tumor grade (*r* = 0.621, *P* < 0.01), in agreement with other malignancies [5, 6, 18]. GFAP is a 55 kDa intermediate filament protein highly expressed in astrocytes of the central nervous system. The least differentiated glioma cells exhibited the lowest GFAP level. Previous studies demonstrated that it could be used as indicators of astroglial differentiation. In the present study, we also found a negative correlation between DcR3 and GFAP expression (*r* = −0.489, *P* < 0.01), which further indicated the close relationship between DcR3 level and the tumor differentiation.

PCNA was originally identified as an antigen that is expressed in the nuclei of cells during the DNA synthesis phase of the cell cycle. The Ki-67 antigen can be exclusively detected within the cell nucleus during interphase, whereas in mitosis most of the protein is relocated to the surface of the chromosomes. Ki-67 protein is present during all active phases of the cell cycle (G1, S, G2, and mitosis) but is absent from resting cells (G0). Both of PCNA and Ki-67 are excellent biomarkers to determine the growth fraction of a given cell population. The fraction of positive tumor cells (the labeling index) is often correlated with the clinical course of cancer. In the current study, we found that LIs of Ki-67 and PCNA rose with the increase of pathology grade. Meanwhile, positive correlations were found between DcR3 expression and Ki-67, PCNA Lis; that is, in the higher actively proliferated gliomas, the positive expression of DcR3 showed stronger expression, which indicates that DcR3 is closely related to the proliferation of glioma cells. Further *in vitro* and *in vivo* studies are needed to investigate the molecular mechanisms of DcR3 influencing the malignant phenotypes of gliomas.

We also attempted to explore the impact of DcR3 on patient survival. Among the 76 cases with follow-up, we found that the survival of the DcR3 negative group was slightly longer than that of the DcR3 positive group (50 ± 1.79 versus 48.36 ± 2.90 months). However, this difference did not reach the significant criterion. The possibility for DcR3 to act as prognostic biomarker for gliomas needed further investigation. For instance, more evidence should be obtained with a cohort with a bigger number of subjects investigated.

## 5. Conclusions

Together with previous studies, the current findings further confirm the role of DcR3 as an oncogene during the tumorigenesis and deterioration of human glioma. DcR3 expression in FFPE samples might be a prognostic biomarker for the differentiation and proliferative status of glioma cells. However, a larger cohort is warranted to verify the evidence. Further *in vitro* and* in vivo* studies are planned to explore the contribution and mechanism of DcR3 in the malignant phenotype of human glioma cells.

## Figures and Tables

**Table 1:** Relationship between DcR3 expression and glioma differentiation.

Parameters	Expression of DcR3 *n* (%)			
	−	+	++	+++
Tissue				
Normal brain (*n* = 18)	16 (88.9%)	2 (11.1%)	0 (0.0%)	0 (0.0%)
Glioma (*n* = 125)	26 (20.8%)	48 (38.4%)	32 (25.6%)	19 (15.2%)
Grade				
Low (*n* = 49)	22 (17.6%)	20 (16.0%)	7 (5.6%)	0 (0.0%)
High (*n* = 76)	4 (3.2%)	28 (22.4%)	25 (20%)	19 (15.2%)

PExpression of DcR3 in normal brain versus low-grade, normal brain versus high-grade, and low-grade versus high-grade: *P* < 0.01.

**Table 2:** Relationship between LIs of Ki-67, PCNA, and pathological parameters $\left(\overset{-}{x}\pm s\right)$.

Parameters	Ki-67	PCNA
Tissue		
Normal brain (*n* = 18)	0.09 ± 0.04	0.04 ± 0.02
Glioma (*n* = 125)	13.59 ± 1.16	11.8 ± 1.02
Grade		
Low (*n* = 49)	1.40 ± 0.21	1.15 ± 0.17
High (*n* = 76)	21.45 ± 1.23	18.73 ± 1.09
DcR3 expression in glioma		
−(*n* = 26)	3.29 ± 1.04	2.78 ± 0.89
+(*n* = 48)	13.63 ± 2.04	11.36 ± 1.70
++(*n* = 32)	15.72 ± 2.06	13.98 ± 1.89
+++(*n* = 19)	24.00 ± 2.06	21.84 ± 1.88

PLIs of Ki-67 and PCNA in normal brain versus low-grade, normal brain versus high-grade, and low-grade versus high-grade: all *P* < 0.01. Pairwise comparisons of Ki-67 and PCNA LIs between DcR3 (−), (+), (++), and (+++): all *P* < 0.05.

**Table 3:** Relationship between DcR3 expression and survival $\left(\overset{-}{x}\pm s\right)$.

DcR3 expression	Survival (*n*)	Dead (*n*)	Survival time (month)
−	19	1	50 ± 1.04
+	28	5	45.26 ± 3.36
++	17	1	54.69 ± 3.39
+++	12	3	26.29 ± 3.74

Total/average	66	10	49.69 ± 2.45
